# The Mental Side of the Sense of Hearing

**Published:** 1898-03-05

**Authors:** 


### THE MENTAL SIDE OF THE SENSE OF HEARING.

The *Laryngoscope* draws attention to the great difference which exists between the capacity of different individuals for appreciating the various qualities of sound, as shown by the tests applied to telephone operators. It has been found that quantitative tests alone are illusory since the inability of the operator to detect the quality of sound is often the cause of delay and confusion. The difference between two operators may in fact be essentially a psychological one, for although the acuteness of hearing of each may be equal the mental processes of one operator may be infinitely more rapid and accurate than those of the other. A simple test sometimes applied is as follows: The superintendent has his own telephone connected simultaneously with those of a number of operators, and calls out, at shorter or longer intervals between each, a written series of numbers, without repetition. These the operators record on paper and the lists are then compared with the original. This is a criterion which clearly tests the various faculties involved. Unfortunately it would seem to complicate the personal factor of the individual with that of the instrument engaged.

